# Therapeutics

**Published:** 1878-03

**Authors:** 


### THERAPEUTICS.

SOME POINTS IN THE ART OF PRESCRIBING FOR CHILDREN.—Robert Farquharson, M.D., F. R. C. P. (*British Med. Journ.*, Sept. 29, 1877.)

I venture to put before you a few practical observations on some points in the art of prescribing for children, because the subject is one which has hardly yet been treated on a sufficiently comprehensive basis. Much valuable but scattered information may be gleaned from the pages of contemporary literature, and much of what I am about to say has been said before; but it seems to me that some little service may be rendered by weaving these threads of knowledge into something of a more connected whole, and obtaining the opinion of some of those experienced physicians who have devoted themselves to the diseases of the very young.

Time, however, will not permit me to do more than touch, and that briefly, upon one point in connection with a subject which is really a large one, and to lay before you some facts and ideas on dosage; and here, again, I must once more subdivide, and take only a small section of a great therapeutical question, whose importance has only very recently begun to acquire that general appreciation which it eminently deserves. I might well be tempted to invite you to join with me in some reflections as to the comparative efficacy of the occasional large or the oft-repeated minute dose—a question which must before long become one of the most pressing in the materia medica; or it might be interesting to inquire as to the desirability or otherwise of inducing the physiological effects of drugs for the relief of pathological conditions; but at this time I mean to restrict myself simply to this proposition—the difference between children and adults in respect of the quantities of various drugs which may be taken, not only with actual impunity, but with absolute benefit.

Now, systematic works have too often not only ignored the teachings of Ringer, Fuller, and other modern investigators, but have done much to hamper and confuse our knowledge in this direction by laying down the law that children necessarily require much smaller doses of most of our active drugs than adults; and we, therefore, see in books on materia medica, as well as on children's diseases, elaborate tables setting forth the quantities to be prescribed with safety at different periods of early life. Some years ago, and possibly even now, a student would run a good chance of being afforded the opportunity of continuing his studies, were he to tell his examiners that a child can take a dose of belladonna with impunity which would probably induce physiological symptoms in the adult; and, as a natural consequence of this mode of teaching, great timidity in practice has resulted; and that this may be a positive evil requires but little reflection to show. If a dose of a particular remedy be too small to effect the purpose for which it is ordered, it is much more likely to do harm than good. Thus an insufficient purgative merely irritates the patient's bowels without giving relief; too small an opiate excites the nervous system and banishes that sleep which it was intended to attract, and numerous other instances will readily occur in illustration of a statement which hardly requires such confirmation.

Granted, then, the importance of administering our remedy in doses sufficient to produce their full remedial effect, I shall lay down, as my first and only proposition, that childen require doses of many medicines quite as large as those which are commonly ordered for persons of mature age. Now, when I speak of children, I shall not refer to mere infants, whose tender organization and sensitive organs and functions require special consideration from a therapeutical point of view. Thus the yielding nature of their skulls, admitting as it must, of wide differences in the proportion of cerebral blood, no less than the natural tendency to sleep at that early age, plainly indicate caution in the use of narcotics. Purgatives and various other remedies must then be used with caution, or we may initiate an irritible condition of stomach and bowels which all our skill may not readily remove. In dealing with general principles, therefore, let it be understood that I refer to children over one year in age, and, perhaps, before beginning the consideration of special instances in favor of my views, I may briefly touch upon the explanations which most naturally suggest themselves of the particular, which forms the excuse for my remarks. In prescribing for adults, we are frequently annoyed by the very various results obtained in different persons from a precisely identical quantity of a peculiar drug. Thus, one patient will develop a copious crop of acne from a few grains of bromide of potassium, whilst another can take ounces without such effect. Another will be salivated by a small quantity of mercury, or be unable to swallow quinine without uncomfortable nervous symptoms or a specially irritable rash. Children, however, do not present, in anything like the same degree, these special peculiarities of idiosyncrasy ; the effects of medicines are pretty constant in their case and we may generally anticipate the satisfaction of finding that our remedy has acted as we wished, and without any of that excess or eccentricity of action which too often brings undeserved discredit on the medical man. The reason which tells us why young children bear heavy doses of potent medicines must also cover this difference from their elders, and we might at once shut up further inquiry by concealing ourselves behind the dense cloak of ignorance implied in the assumed fact of an ultimate difference of constitution. But, true as this may be as an abstract proposition, we must look a little deeper, and ask, in the first place, whether some peculiarity of digestion may not come to our aid, and whether infants may not emulate some of the lower animals in the power which they possess of neutralizing or destroying poisonous principles, as rabbits harmlessly browse on belladonna, and pigeons baffle the deadly action of strychnia, etc. But of such powers in the human being, at any period of life, we have no shadow of proof, presumptive or otherwise ; and it is probable that remedies reach the blood of children in the regular way, and through the same chain of physiological processes as in the case of adults. So we must again go forth in search of our explanation ; and I think we may find some approach to it, at all events, in the view that, in consequence of the rapid growth taking place in the body during early life, the blood and tissues are in a condition of specially active destruction and renovation. Drugs such as the metals, which probably combined with the albumen of the circulating fluid, are here rapidly cast out of the system. Other remedies, which act more particularly on the nervous system, are cast out with effets matters before they have had full time to produce their physiological effects, or, at all events, before these effects have attained to anything like completeness. Thus we do not often find developed in children that accumulation which occasionally, if rarely, is observed in patients of older gowth, because the drug is removed before it can produce that continuous and ever intensifying influence on the nervous system which eventually finds expression in what we may call a discharge.

So much, therefore, for my explanation, such as it is, of the facts which I shall now proceed briefly to lay before you.

Now, in the first place, I am bound, of course, to confirm the usual opinion of the dangers of opium in very early childhood ; and it is not long since I saw an infant of eight months nearly narcotized to death by six two-minim doses spread over two days. But those within the period of life which I have selected for consideration can bear moderate quantities, and chloral seems always well borne. For instance, I have lately had under treatment a little rickety girl suffering from recurring attacks of laryngismus stridulus, to whom three and a half grains were given with benefit thrice daily. The same patient took ten, and finally fifteen grains of bromide of potassium, before any beneficial effect was attained ; and I have always observed that this drug is well taken by children. Twenty to thirty grains have been no uncommon dose to reach in patients of from eight to ten suffering from epileptic seizures, and in them I have never observed any symptoms of bromism. The opposite seems to hold good of potassium iodide, so far as my limited experience goes; for I have three times seen papular and petechial eruptions produced by one-grain doses of this drug, and I should specially like to ask whether this corresponds with the observation of others.

Arsenic is usually well taken. I should have no hesitation in ordering five minims of Fowler's solution for a child of six years old. Ten minims have been occasionally ordered ; and I had recently under care a little girl, aged ten, whose somewhat obstinate psoriasis only begun to yield when the dose was pushed up to sixteen minims. When physiological symptoms present themselves, as they sometimes do, it is important to know that they do not assume the usually described type, and that vomiting is the most usual symptom. I have seen this follow a single one-minim dose, and more rarely we meet with a red and irritable tongue, dry lips, injected eyes and abdominal pain ; girls being in my experience, contrary to the statement of Ringer, more susceptible to the overaction of the drug than boys.

Prussic acid may be pretty freely prescribed, and I have given nearly two minims to a child of two years, with some slight benefit, for pertussis ; and at the age of seven I have given nearly three minims for the successful arrest of sickness.

We know that emetics must be given in very full doses. The intestinal canal of young children seems strangely insusceptible to the action of purgatives, and large quantities of Gregory's and compound jalap powders must be given before satisfactory action is attained.

I have by no means exhausted the instances to be gleaned from my own experience or that of others in support of my main proposition ; but time presses, and I will conclude with a reference to belladonna, whose comparative harmlessness to young children has been most amply confirmed since Fuller first pointed out the fact some years ago. I have very commonly prescribed from 20 to 30 minims of the tincture for children of from 15 months to 5 years, and have invariably found that the younger the child the less likely was the dose to be followed by physiological symptoms. I have on several occasions pushed the quantity up to one and a half and even two drachms of the tincture three times a day, in children of from 10 to 12, with only a very tardy development of uncomfortable results ; but, in my experience, a few 10-minim doses are usually sufficient to cause uncomfortable dryness of the throat in adults. In children, however, we seldom have complaints of this, nor do we observe dilatation of the pupil ; general languor, want of appetite, troublesome diarrhœa, perspiration about the head and rapidity of pulse, being in them usually obscured.

I have ventured to bring these few remarks before you, as the outcome of some little observation and experience, and in the hopes of stimulating discussion on a subject which seems to afford a promising field for future investigation.

